# Relational Ecological Momentary Assessment: A Viewpoint on the Relational Decision Space in Digital Mental Health

**DOI:** 10.2196/99699

**Published:** 2026-11-13

**Authors:** David Jonas Fisher, Lars Rossen

**Affiliations:** 1 Independent Researcher, Buegangen 3, Fanø, 6720, Denmark, 45 30480745; 2 Independent Researcher, Varnæs, Denmark

**Keywords:** relational decision space, relational digital health, ecological momentary assessment, just-in-time adaptive intervention, dyads, relational support, receptivity, support eligibility

## Abstract

Digital mental health has become increasingly capable of representing and adapting to individuals in time. When support is delivered through another person, however, the relevant decision context includes conditions that cannot be specified from the primary user alone. This viewpoint proposes making selected conditions relevant to a support decision within a specified relationship explicit. We introduce relational ecological momentary assessment (REMA) as a conceptual method for representing these conditions in a relational decision space. In its initial dyadic implementation, REMA combines a low-burden report of the primary user’s emotional zone (Z) with supporter contact availability (Cp) and supporter capacity (Cs). The meaning of these reports depends on the relationship and the proposed form of support. Willingness, consent, and safety determine whether an action is permissible to consider, while data validity concerns whether the reports are current and interpretable. These conditions and rules delimit relational support eligibility by specifying which classes of support can still be considered. Coregulation as a reciprocal temporal process remains outside the scope of this implementation. We situate the proposal within research on ecological momentary assessment, just-in-time adaptive interventions, dyadic digital health, and systemic digital mental health; provide a hypothetical implementation example; and show how 1 Z report can inform separate decisions involving different supporters. REMA remains conceptual and requires empirical evaluation. Its broader proposition is that when digital health recruits another person, the decision-relevant conditions across that specified relationship should be made explicit.

## Introduction

Digital mental health has made substantial progress in bringing assessment and intervention closer to everyday life. Ecological momentary assessment reduces reliance on retrospective recall by capturing experience near the moment of occurrence, ecological momentary interventions extend support into natural settings, and just-in-time adaptive interventions (JITAIs) provide a design language for adapting support to changing conditions [1-3]. These developments have changed how mental health can be measured and supported over time. However, the individual remains the dominant unit of measurement, decision, and intervention. Even when social context is acknowledged, relationships often enter as background variables, perceived support measures, communication channels, or downstream outcomes rather than as an explicit decision-relevant layer.

That individual-centered architecture is appropriate for many problems, but it becomes incomplete when the proposed intervention itself depends on another person. Psychological strain and recovery can unfold through attachment, coregulation, partner responsiveness, family synchrony, caregiving, misattunement, and repair [4-11]. In everyday support, the practical question is not only how the primary user feels. It is also whether a particular supporter can be reached, whether contact with that person is wanted, whether the supporter has enough capacity to engage without predictable overload, and whether a well-intended attempt could intrude or intensify strain.

Dyadic and family digital health interventions increasingly involve supporters, partners, parents, and family members [12-14]. Dyadic digital health work has explicitly argued for intervening at the level of 2 interdependent people, while recent systemic digital mental health scholarship argues that multimember interventions require design assumptions that differ from simple translations of individually oriented tools [13,15]. These developments move the field toward relational design, but participation by more than 1 person does not by itself specify the momentary conditions governing action between them. A platform may enable communication without specifying when contact should be deferred, a dyadic intervention may deliver parallel content while leaving supporter capacity implicit, and a dashboard may make information visible without making the relational conditions for action inspectable.

This viewpoint advances a broader proposition: when health-related action depends on another person, the decision context extends beyond either participant alone. We use “relational decision space” to mean a representation of selected current conditions relevant to a support decision within a specified relationship. The relationship and the proposed form of support provide the context for interpreting these conditions.

Relational ecological momentary assessment (REMA) is offered as a conceptual method for making these conditions explicit. Its initial dyadic implementation represents current conditions on both sides of a specified relationship, distinguishes momentary reports from permission and data validity, and uses this information to constrain relational support eligibility—that is, which classes of support can still be considered under the stated conditions and rules. This viewpoint presents the conceptual method and its proposed empirical evaluation.

## From Individual Adaptation to Relational Representation

JITAI science distinguishes moments of vulnerability from moments of opportunity and increasingly emphasizes that intervention timing also depends on receptivity and engagement [3,16]. Recent work further identifies the limited incorporation of social relationships into JITAIs as an important research gap [16]. This matters because the opportunity to deliver a self-directed intervention and the opportunity to mobilize another person are not the same decision problem. Once another person becomes part of the intervention, opportunity and receptivity can be distributed across participants: a primary user may be highly vulnerable, while the relevant supporter is unavailable, depleted, unwilling to engage, or inappropriate for the form of support under consideration.

Emerging social-support JITAI research makes these constraints concrete. Elmer et al [17] compared triggering strategies in a feasibility study for people with depressive symptoms and found that interventions triggered by self-reported momentary need for support were perceived as better timed and more helpful than interventions triggered by distress indicators; actual support seeking followed only a minority of triggered interventions, and perceived unavailability of support providers was one barrier. Their design also limited intervention delivery and asked participants to identify both the type of support needed and an appropriate person in their network [17]. These findings illustrate why decisions about seeking support also need to account for the circumstances of the person being approached.

REMA adds a relational layer to individual adaptation. Alongside “What intervention should this individual receive now?,” it asks “What is currently possible through this specific relationship?” The decision concerns a defined form of support within a specified relationship. Joint reports inform that decision, and their relevance depends on the relationship and the proposed support. Relational support eligibility is one answerable question within this representation: which classes of action are not ruled out by current conditions, mutual permission, and valid information? The primary user’s emotional zone (Z), supporter contact availability (Cp), and supporter capacity (Cs) provide an initial low-burden description of conditions for that decision.

## REMA as a Method for Informing Relational Decisions

REMA begins with 1 primary user and 1 supporter in a specified relationship. These are functional roles within a support moment. This dyad is the starting unit for the proposed support decision: conditions on both sides can alter whether and how support can proceed.

The initial dyadic implementation uses 3 momentary inputs. Z represents current regulatory demand using 3 broad levels: calm, alert, and activated. These categories are intended for coarse self-report, and their validity, thresholds, and usefulness require empirical testing. Cp represents whether the channel for contact with that primary user is currently open, partially constrained, or blocked; the initial REMA specification uses 4 ordered levels ranging from open to blocked. Cs represents the supporter’s current ability to engage without predictable overload, likewise using 4 ordered levels from high capacity to depleted.

Z, Cp, and Cs describe selected momentary conditions for the decision. With 3 Z levels and 4 levels each for Cp and Cs, the initial classification yields 48 combinations (3 ×4×4) for empirical evaluation. Within a specified relationship and support context, the same Z can leave different actions open when Cp or Cs changes, and the same supporter reports can have different implications as Z changes. Cp is specific to contact with the primary user, and Cs is a momentary capacity report; willingness to help is considered separately.

The initial inputs are deliberately limited. Relationship history, attachment orientation, conflict intensity, cultural context, coercive dynamics, role expectations, and contextual stressors can shape what contact, availability, and capacity mean in a particular relationship. Additionally, 2 partners and 2 former partners could report identical Z-Cp-Cs values, yet “available for contact” could refer to emotional support in 1 relationship and practical arrangements in the other. Requiring all of these contextual factors as repeated momentary inputs would increase burden and could undermine everyday use; engagement and attrition are already central challenges in digital interventions [18,19]. REMA therefore starts with a low-burden set of inputs whose adequacy needs to be examined in the intended setting.

Table 1 separates the conditions REMA represents for a support decision from the momentary reports and from the later layers of admissibility, data validity, eligibility, and final selection.

**Table 1.** Conceptual layers and scope of the dyadic method.

Layer	What it represents	Decision role	What it does not imply
Relational decision space	A representation of selected current conditions relevant to a support decision within a specified relationship	Makes selected conditions explicit for that decision	A complete model of relationship quality, history, reciprocity, or coregulation
Momentary state information	Z: primary user’s emotional zone, Cp: dyad-specific supporter contact availability, and Cs: supporter capacity	Provides momentary information for the specified support decision	That the joint reports constitute the relationship
Admissibility	Primary-user contact preference, supporter willingness, and consent and safety constraints	Can veto or narrow relational action before eligibility is considered	A prediction that support will be helpful
Data validity	Whether required information is current, missing, expired, uncertain, or discordant	Determines whether the decision context is sufficiently intact	Permission to infer unreported states
Eligibility	Which bounded support classes are not ruled out by the preceding constraints	Produces an eligible response set, including the possibility of no relational prompt	A clinical recommendation or final intervention choice
Final selection	Choice among eligible options, if more than 1 remains	A separate implementation and empirical problem	An empirically validated optimal selector
Network extension	Conditions for separate support decisions involving different supporters	Shows how dyadic decisions could inform a broader relational architecture	A fully specified one-to-many allocation algorithm

## Admissibility, Receptivity, and Consent

Availability and capacity are necessary but not sufficient conditions for relational support. Receptivity must be considered on both sides of the dyad and may be specific to the proposed form of support [16]. A primary user may want acknowledgment but not discussion or may want space despite high distress. A supporter may be reachable and sufficiently resourced but unwilling to engage in a particular type of interaction at that moment.

REMA therefore treats willingness and consent as admissibility conditions rather than as additional dimensions multiplied into the momentary state space. Before a relational support class can be considered, the relevant preferences and permissions must not rule it out. A request for no contact should override an otherwise favorable state configuration. Likewise, supporter refusal or withdrawal of consent should block routing to that supporter. Unknown willingness should not be treated as affirmative consent.

Z, Cp, and Cs provide momentary information for the support decision. Admissibility rules determine whether relational action is permissible to consider; data validity rules determine whether the information is sufficiently current and interpretable. Eligibility logic narrows the set of support classes that can still be considered. Final selection, where needed, is a separate implementation and empirical problem. REMA’s contribution occurs earlier in the chain: it makes selected conditions and their implications for action explicit and open to challenge.

## From Reported Conditions to Inspectable Decisions

REMA uses deterministic language only for the rule layer. Given a stated set of classified inputs, validity requirements, and admissibility conditions, a system should be able to show why a relational action class remained eligible for consideration, was blocked, or was deferred. The computation applies predefined distinctions and rules; the relational meaning of the reports must be established within the specified relationship and support context.

The method therefore emphasizes transparent rules at this early stage. A relational recommendation can impose costs on someone other than the primary user, create unwanted contact, or repeatedly recruit the same supporter. In such settings, inspectability is part of ethical accountability [20,21]. Probabilistic or adaptive components may later improve timing or personalization, but explicit consent, safety, and burden constraints should remain visible.

Figure 1 summarizes the operational sequence. Primary-user and supporter reports inform a support decision within a specified relationship, data validity and admissibility constrain relational support eligibility, and eligible action classes can then enter a separate selection step. The lower panel shows the conceptual one-to-many extension: 1 Z report may inform separate decisions involving different supporters, while network allocation requires additional rules.

**Figure 1.**
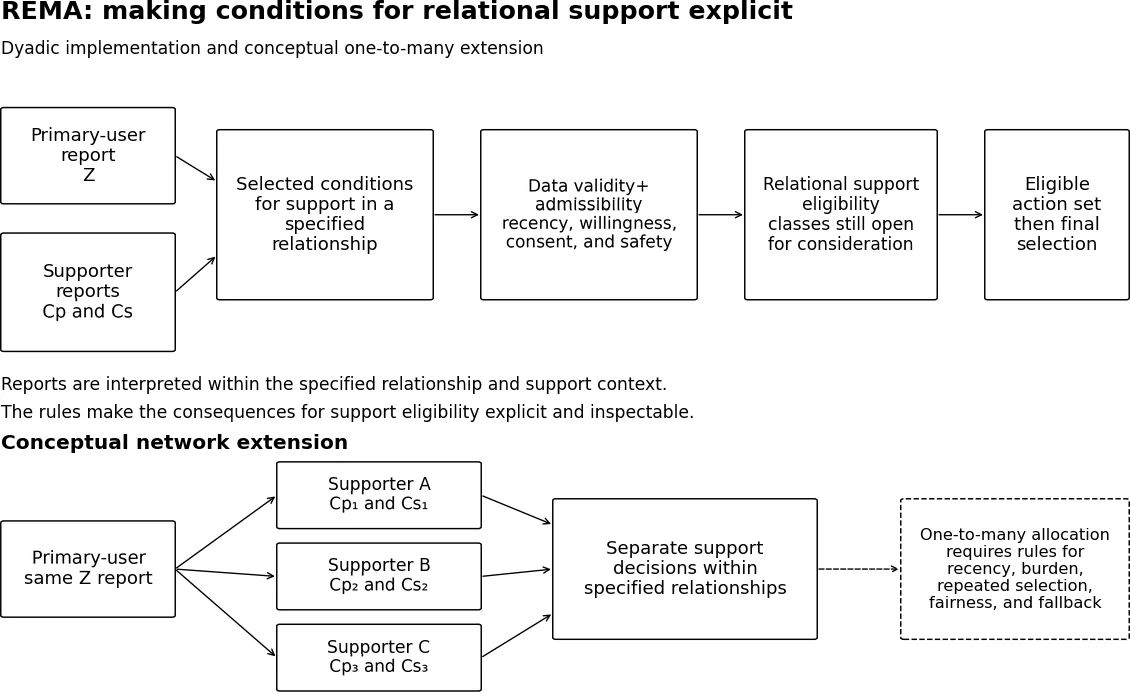
Relational ecological momentary assessment (REMA) as a method for making selected conditions for relational support explicit. The dyadic flow distinguishes the representation of selected conditions, the validation and admissibility checks of the data, the determination of eligible support classes, and the subsequent final selection within a specified relationship and support context. The conceptual network extension illustrates separate decisions involving different supporters; formal one-to-many allocation requires additional rules. Cp: supporter contact availability; Cs: supporter capacity; Z: primary user’s emotional zone.

## A Stable Set of Bounded Outputs

REMA’s proposed outputs are classes of low-intensity support. The proposed set comprises grounding, boundary protection, low-demand communication, deferred-contact planning, and relational repair. Grounding supports brief stabilization or orientation when activation is high. Boundary protection reduces relational demand when capacity or safety is limited. Low-demand communication permits brief, concrete contact when support is possible but should remain light. Deferred-contact planning makes postponement explicit rather than leaving silence ambiguous. Relational repair refers to low-intensity reattunement after manageable strain and requires mutual willingness.

Two additional terms describe how the system operates. “No relational prompt” is a system-level decision when relational action is inadmissible, unnecessary, or insufficiently supported by current information. “Uncertainty” is a decision mode triggered by inadequate information. Self-regulation may be supported through grounding or external resources when relational action is unavailable; this does not place responsibility for resolving relational strain on the primary user alone.

Table 2 gives illustrative examples of how reported conditions may constrain these classes. A complete 48-combination mapping would require empirical calibration, version control, and context-specific safety rules.

**Table 2.** Illustrative constraints on bounded support classes.

Illustrative condition	Admissibility and data status	Eligible direction	Reasoning
Z^a^ reported as calm; supporter available with adequate capacity	Contact wanted and information current	No relational prompt or optional low-demand contact	Stable conditions do not require intervention
Z reported as alert; partial availability and moderate capacity	Brief contact wanted	Low-demand communication or grounding	Demand should remain proportionate to capacity
Z reported as activated; supporter available but limited capacity	Contact wanted; information current	Grounding; possibly brief copresence	High activation should not automatically increase relational demand
Any Z; supporter contact blocked	Relational channel unavailable	Deferred-contact planning or no relational prompt	Availability constrains action regardless of primary-user need
Any Z; primary user requests space	Contact inadmissible	No relational prompt; self-regulation support may remain available	Willingness overrides an otherwise favorable state configuration
Supporter information expired or missing	Decision context incomplete	Lower-demand or nonrelational options; refresh before demanding contact	Unknown capacity must not be inferred as available capacity

a Z: primary user’s emotional zone.

## A Minimal Implementation Example

A hypothetical implementation illustrates how the method could work. Consider a low-burden system in which the primary user can check in on a scheduled or user-initiated basis and indicate both current Z and whether relational contact is wanted. Supporter Cp and Cs can be updated on a similarly low-burden basis but need not be solicited continuously. In some implementations, supporter input could instead be requested when the primary user indicates a desire for relational support, reducing unnecessary reporting. The appropriate strategy is an empirical design question and may differ by population. The system uses current reports from both participants to inform the support decision.

Suppose the primary user selects the alert category after a difficult exchange and wants only brief acknowledgment. The supporter has recently reported partial availability and moderate capacity and remains willing to provide low-demand contact. Under those conditions, emotionally demanding repair would be ruled out, while a brief acknowledgment or deferred plan could remain eligible. If the primary user instead requests space, relational contact is inadmissible regardless of Cp and Cs. If the supporter’s information has expired, the system should not infer continuing availability or capacity; relationally demanding outputs should narrow or be withheld until information is refreshed.

Sampling frequency would depend on the setting. Elmer et al [17] limited intervention opportunities in their social-support JITAI, while other contexts may favor event-contingent or hybrid sampling. Reports should be recent enough for the use case and collected with minimal burden and user control. Decisions should become more conservative when information quality deteriorates.

## Missing, Expired, Uncertain, and Discordant Information

Data problems should not be collapsed into 1 generic category because they carry different implications. Missing information means that a required report is absent. Expired information was previously available but has exceeded a prespecified validity window. Uncertain information is present but not sufficiently reliable or interpretable for a confident decision. Discordant information occurs when reports or preferences conflict in ways that matter to the proposed action.

A REMA-like implementation should specify validity windows appropriate to the population and use case rather than assume 1 universal duration. Expired Cp or Cs should not be carried forward as current. Missing supporter information should not be interpreted as availability, and missing primary-user information should not license demanding relational action. Uncertainty should generally narrow the action set. Discordance should not be silently averaged away; where the conflict concerns consent, safety, or contact preference, the more restrictive condition should govern until clarified.

As information becomes less reliable, the system should narrow or withhold relational prompts. Depending on the circumstances, that may mean a lower-demand class, deferred contact, a nonrelational self-regulation option, or no relational prompt.

## Safety and Boundary Conditions

REMA is intended for low-intensity, consent-sensitive support in settings where both parties can report with sufficient autonomy for the decision context to remain meaningful. Safety is therefore a positive design requirement: the architecture must preserve opt-out, boundary protection, conservative handling of uncertainty, and explicit limits on what the system is authorized to encourage.

Relational prompting may be inappropriate where relationships are coercive, violent, chronically unsafe, strategically manipulative, or characterized by severe power imbalance. It is also inappropriate as a substitute for crisis services, suicide risk assessment, professional clinical judgment, or psychotherapy. Real-world research would require setting-specific screening, adverse event monitoring, escalation pathways, privacy safeguards, and clear explanation of system limits.

Several failure modes are empirically important. False eligibility occurs when minimum constraints fail to block support that should have been deferred or further assessed. False blocking occurs when potentially useful support is withheld. Burden underdetection occurs when a supporter appears available while cumulative demands are depleting capacity. Stale-state dependence occurs when outdated information is treated as current. These failure modes should be measured during empirical evaluation.

## Scope and Boundary of the Relational Claim

The present method makes selected conditions relevant to a specified support decision explicit. It distinguishes Z, Cp, and Cs from admissibility and data validity rules and then uses these layers to determine which support classes remain eligible. Relationship quality, attachment, historical reciprocity, trust, conflict patterns, and power can shape the meaning of the reports and the appropriateness of the proposed support. Their relevance must be considered when defining the support context and interpreting the reports, while the initial momentary inputs remain Z, Cp, and Cs.

Coregulation provides important theoretical context but is outside the scope of the present dyadic implementation. It is reciprocal and temporal: each person’s state and behavior can alter the other’s over time [6,7]. The initial dyadic implementation is asymmetric and cross-sectional at each decision point, although repeated observations can later be analyzed longitudinally. A reciprocal extension would require role switching, mutual state representation, interaction history, and temporal rules beyond the present formulation.

A complete set of rules for allocating support across multiple relationships also remains outside the present dyadic implementation.

## From Dyads to Relational Networks

The dyadic implementation provides a starting point for considering multiple relationships. A primary user may be embedded in several simultaneously relevant relationships, so 1 Z report can coexist with different Cp, Cs, willingness, and data validity conditions for different supporters. The primary user’s report is unchanged, but the possibilities for support differ across relationships. Each support decision therefore requires information about the relevant relationship.

Consider a primary user reporting the alert category and 3 potential supporters. One may be reachable and have adequate capacity; a second may be reachable but depleted; a third may have capacity but be unavailable for this relationship, unwanted for the relevant form of contact, or represented only by stale information. These relationships present different conditions for a support decision. A relational architecture should preserve those distinctions when considering whom to involve.

A one-to-many architecture therefore raises a further question: through which relationship, if any, should an eligible action proceed? This would require rules for recency, accumulated burden, repeated selection, fairness or rotation, competing eligible relationships, fallback when no dyad is viable, and role-specific constraints. The dyadic representation provides a starting point for developing these allocation rules.

## Future Empirical Agenda

The scientific value of REMA depends on empirical evaluation rather than conceptual plausibility. Initial work should test feasibility and burden: whether primary users and supporters can provide the minimal inputs with acceptable friction; whether willingness reporting is understandable; how often data become missing or stale; and whether people experience the representation of relational conditions as respectful, useful, intrusive, reductive, or irrelevant.

Measurement studies should examine whether Z, Cp, and Cs are reliable enough for their intended decision roles and whether their categories correspond to meaningful differences in distress, perceived support, relational strain, supporter burden, or contact success [22-27]. The categories remain design hypotheses. Their thresholds, labels, and number of levels may need revision or collapse after empirical study. Equally important, research should test whether the combined representation captures decision-relevant relational information above and beyond primary-user state alone.

Subsequent studies should test decision validity rather than assume it. Researchers could compare REMA-constrained decisions with clinician judgment, participant preference, or alternative rule sets; examine false eligibility and false blocking; and evaluate whether explicit admissibility rules reduce unwanted contact. Micro-randomized trials could later test proximal effects of eligible low-intensity support classes under defined conditions [28]. Analyses should account for dyadic interdependence, asynchronous reporting, temporal windows, within-dyad clustering, and missingness. Network studies should additionally examine whether conditions across multiple relationships can be represented without excessive burden and whether coordination rules distribute demands safely and acceptably across supporters.

A further question is comparative value. Does explicitly representing these decision conditions improve trust, perceived fairness, safety, or error detection compared with systems that infer support opportunities primarily from the primary user’s state? Does supporter input add enough decision value to justify its burden? More fundamentally, does representing relational conditions explicitly improve the design of digital interventions that recruit another person? Implementation research should also examine whether such a layer can be integrated into scalable digital mental health services without disproportionate workflow or participation costs [29].

## Conclusions

Digital mental health has become increasingly skilled at representing and adapting to individuals over time. When health-related action depends on another person, individual state alone cannot specify the full decision context. Current conditions across the relationship can shape whether contact is possible, wanted, and manageable and whether the available information is sufficient to consider it.

REMA proposes representing selected conditions relevant to a support decision within a specified relationship. It offers a conceptual method for making these conditions visible and inspectable and then using validity and admissibility constraints to delimit relational support eligibility. The Z-Cp-Cs representation is an initial way to record these conditions. These reports must be interpreted within the specified relationship and support context.

Whether REMA’s present inputs are valid, whether its constraints improve decisions, and whether eligible support classes improve outcomes are empirical questions. The methodological proposition is that when digital health recruits another person, the decision-relevant conditions across that specified relationship should be made explicit. The dyad provides the starting case; multiple simultaneous dyads point toward a broader relational architecture. If supported empirically, this principle could inform relational digital health technologies in which decisions about support explicitly account for conditions across the relevant relationship.

## Abbreviations

JITAI: just-in-time adaptive intervention
REMA: relational ecological momentary assessment
